# Evolutionary Analysis of *StSnRK2* Family Genes and Their Overexpression in Transgenic Tobacco Improve Drought Tolerance

**DOI:** 10.3390/ijms24021000

**Published:** 2023-01-05

**Authors:** Panfeng Yao, Lei Sun, Simon Dekomah, Zhenzhen Bi, Chao Sun, Juan Mao, Chunli Zhang, Tianyuan Qin, Yihao Wang, Yuhui Liu, Zhen Liu, Kazim Ali, Jiangping Bai

**Affiliations:** 1 State Key Laboratory of Aridland Crop Science, Gansu Agricultural University, Lanzhou 730070, China; 2 College of Life Science, Sichuan Agricultural University, Ya’an 625014, China; 3 College of Agronomy, Gansu Agricultural University, Lanzhou 730070, China; 4 College of Horticulture, Gansu Agricultural University, Lanzhou 730070, China

**Keywords:** potato, abiotic stress, drought, ABA, SnRK2, evolutionary analysis

## Abstract

Sucrose non-ferment 1-related protein kinase 2 (SnRK2) is a highly conserved protein kinase in plants that plays an important role in regulating plant response to drought stress. Although it has been reported in some plants, the evolutionary relationship of potato SnRK2s and their function in drought resistance have not been systematically analyzed. In this study, molecular characteristic analysis showed that 8 *StSnRK2s* were distributed on six chromosomes, coding proteins were divided into three subgroups, and *StSnRK2s* clustered in the same subgroup had similar conserved motifs and domains. In addition, *StSnRK2* has a wide range of replication events in some species, making it closer to dicots in the process of evolution. In addition, the average nonsynonymous substitution rate/synonymous substitution rate (Ka/Ks) value of *SnRK2s* in monocots was higher than that of dicots. The codon usage index showed that *SnRK2s* prefer to use cytosine 3 (C3s), guanine 3 (G3s) and GC content (GC3s) in monocots, whereas thymine 3 (T3s) and adenine 3 (A3s) are preferred in dicots. Furthermore, stress response analysis showed that the expression of *StSnRK2s* under different degrees of drought stress significantly correlated with one or more stress-related physiological indices, such as proline and malondialdehyde (MDA) content, superoxide dismutase (SOD) and catalase (CAT) activity, ion leakage (IL) etc. The drought resistance of *StSnRK2* transgenic plants was determined to occur in the order of *StSnRK2.1/2.8* > *StSnRK2.2/2.5* > *StSnRK2.4/2.6* > *StSnRK2.3* > *StSnRK2.7*, was attributed to not only lower IL but also higher proline, soluble sugar contents and stress-related genes in transgenic plants compared to wild type (WT). In conclusion, this study provides useful insights into the evolution and function of *StSnRK2s* and lays a foundation for further study on the molecular mechanism of *StSnRK2s* regulating potato drought resistance.

## 1. Introduction 

As sessile organisms, plants are often subjected to various adverse environmental factors, such as drought, salinity, low temperature, high temperature, etc. [1]. Among them, drought is the main environmental factor that restricts plant growth and development and causes serious reductions in the yield of all kinds of crops all over the world, even resulting in no harvest in some areas [2]. Cultivating drought-tolerant varieties by manipulating drought-tolerant genes is a potential and attractive measure to address these challenges [3,4]. In this regard, in-depth mining of key genes that play important roles in regulating plant drought resistance and clarification of the molecular mechanisms of their response and adaptation to drought stress are of great significance to alleviate the effects of drought stress on crop yield [5,6].

As an important phytohormone, abscisic acid (ABA) plays an important role in plant response to drought stress [7,8]. There are three core components in the plant ABA signaling pathway: ABA receptors pyrabactin resistance 1/PYR1-like/regulatory components of ABA receptors (PYR/PYL/RCARs), type 2C protein phosphatase (PP2C) and sucrose non-fermenting 1-related protein kinases 2 (SnRK2s), which together form a double-negative regulation system, i.e., PYL-PP2C-SnRK2, to regulate ABA signal transduction [9]. When plants are subjected to drought stress, ABA accumulation increases rapidly. Then, ABA is perceived and bound by its receptors, PYL, and releases SnRK2 by inhibiting the activity of PP2C. Activated SnRK2 kinase can phosphorylate and activate downstream transcription factors (TFs) such as ABA-responsive element-binding protein (AREB)/ABRE binding factors (ABFs) and ultimately improves plant drought resistance [10,11]. In this complex process, SnRK2 plays a pivotal role in transmitting the ABA signal and activating downstream protein expression [12]. Phylogenetic analysis showed that SnRK2 family members can be divided into three subgroups. Group I kinases cannot be activated by ABA, group II kinases can be activated or weakly activated by ABA (depending on the plant species) and group III kinases can be strongly activated by ABA. To date, *SnRK2* genes have been isolated and identified in several species, such as 10 genes in Arabidopsis [13], 10 genes in rice [14], 14 genes in maize [15], 22 genes in soybean [16], 22 genes in tobacco [17], 8 genes in camellia [18], 20 genes in cotton [19], 9 genes in pepper [20], 14 genes in grape [21] and 8 genes in mung bean [22]. 

In recent years, with the gradual resolution of the ABA signaling pathway, remarkable progress has been made with respect to the role of SnRK2 proteins in regulating plant drought resistance. In *Poncirus trifoliata*, ABA-dependent kinase SnRK2.4 was reported to enhance the expression of the *ADC* gene by phosphorylating ABF2 protein, which makes transgenic plants accumulate putrescine, resulting in stronger drought resistance [23]. *Arabidopsis thaliana* SnRK2.4 promoted the expression of *DREB2A*, *DREB2B* and *RD29A* genes through interaction with MYB21 and ultimately improved plant tolerance to drought stress [24]. SnRK2.6 induced stomatal closure by phosphorylating ubiquitin E3 ligase RZFP34/CHYR1, thus enhancing the drought resistance of transgenic plants [25]. SAPK9/SnRK9 has been reported to directly phosphorylate MADS23 in an ABA-dependent manner; this activated MADS23 then directly regulates the high expression of its target genes, *NCED2*, *NCED3*, *NCED4*, and *P5CR*, thus increasing ABA and proline accumulation and ultimately improving the drought tolerance of transgenic rice [26]. In *Triticum aestivum*, SnRK2.8 enhances the drought resistance of transgenic plants by activating ABA-dependent pathway genes (*RD20A* and *RD29B*) and ABA-independent pathway genes (*CBF1*, *CBF2* and *CBF3*) [27]. In *Morus alba*, SnRK2.1/2.4 forms a ternary complex with Gγ1/Gγ2 and PP2Cs, which enhances the adaptability of plants to drought stress by enhancing ABA signaling [28]. These studies indicate that SnRK2 kinase plays a critical role in supporting plant resist drought stress; there are some differences in signaling pathways, but its effects mainly occur through phosphorylation modification to regulate downstream gene expression and protein activity. Therefore, it is highly important and urgent to deeply explore the involvement of SnRK2 kinase in drought resistance and to clarify its molecular mechanism for enhancing plant resistance and improving crop yield under drought stress.

Potato (*Solanum tuberosum* L.) is indispensable to global food security and is the fourth largest staple crop in China [29]; this crop is mainly planted in the northwest of China [30]. Owing to the natural conditions of water shortage in northwest China, potatoes often suffer from drought stress, which seriously affects the local and even national commercial yield of potatoes [31]. Consequently, research on drought resistance genes and functional identification of potato has become a hot spot in recent years. For instance, the overexpression of *StGA2ox1* [32], *StDRO1* [33], *StPIP1* [34], *StRFP2* [35], *StMAPK11* [36] and *StProDH1* [37] genes has effectively improved the adaptability of transgenic plants to drought stress. However, few studies have been conducted on the function of core genes of the ABA signaling pathway in potato response to drought stress. In our previous study, a total of eight *StSnRK2* genes (*StSnRK2.1-2.8*) were isolated, and the subcellular localization of StSnRK2 proteins was analyzed [38]. On this basis, in this study, we further investigated the gene structures and chromosomal distribution of *StSnRK2s*, the conserved motif and evolutionary relationship of coding proteins, selection pressure and codon usage bias using TBtools and related software. In addition, the expression profiles of *StSnRK2s* under ABA and drought stress were detected by qPCR. Finally, the function of StSnRK2 genes in response to drought stress was verified by stable transformation of tobacco. The results of this study provide useful information, contributing to the understanding of the function of *StSnRK2* genes, which are valuable candidates for genetic improvement of drought resistance in potato.

## 2. Results

### 2.1. Gene Structure, Motif Composition and Chromosomal Distribution of StSnRK2 Genes

We first explored the characteristic regions of StSnRK2 proteins, and Arabidopsis *SnRK2* genes were selected as a reference. Ten conserved motifs were identified through the MEME website and visualized in TBtools (Figure S1A,B). The length of these motifs ranges between 11 (motifs 7 and 9) and 50 (motifs 1, 2, 3 and 4) amino acids. The number of motifs in StSnRK2 proteins varies from 3 to 9. The motifs of StSnRK2s are highly conserved, and motifs 1–7 exist in almost all StSnRK2s, excluding StSnRK2.8. With the exception of StSnRK2.8, all other proteins contain at least seven motifs. However, there are some variations between different groups. For example, motif 9 exists only in group I, and motif 8 is unique to group I, except AtSnRK2.9, suggesting that these motifs may play an important role in the gene function of different subfamilies. 

We also clarified the structural composition of *SnRK2* genes. The results show that all the coding sequences of *SnRK2* genes were destroyed by introns, with the number of introns ranging from 6 to 9, demonstrating some differences in degree among the 8 *StSnRK2* genes and 10 *AtSnRK2* genes (Figure S1A,C). The genes with the largest number of introns are *StSnRK2.2*, *AtSnRK2.2* and *AtSnRK2.6*. Interestingly, there are fewer differences in exon/intron structure between different subfamilies of *StSnRK2s*, which may indicate that there is no significant difference in the gene structure of *StSnRK2s* in the process of evolution. 

**Figure 1:**
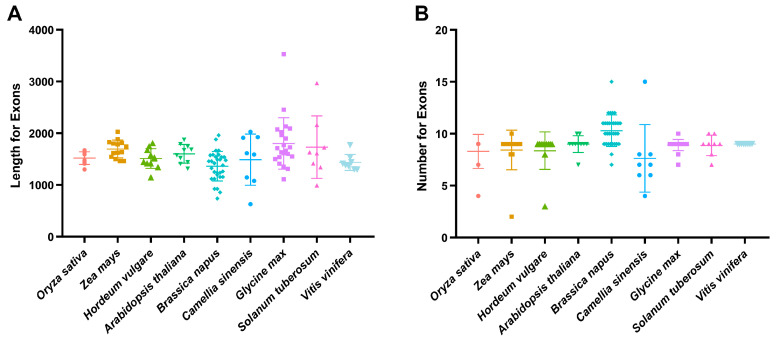
Comparative analysis of exons and introns between monocotyledons and dicotyledons. (**A**) Exon length of *SnRK2* genes in nine species. (**B**) Exon number of *SnRK2* genes in nine species.

Furthermore, the gene structure of *SnRK2* family genes in potato and eight selected species were analyzed. The cDNA length of 122 *SnRK2* genes varies from 627 (*CsSnRK2.18*) to 3528 (*GmSnRK2.7*) bp. The variations among *Brassica napus*, *Camellia sinensis*, *Glycine max*, and *Solanum tuberosum* cDNA length is significant, whereas other species are relatively concentrated (Figure 1A). Moreover, the predicted exon structure shows that 59.8% of *SnRK2* genes harbor nine exons. Notably, *Vitis vinifera* SnRK2 genes have nine exons, indicating a highly conserved gene family. The exon number of *SnRK2* in *Camellia sinensis* varies greatly, ranging from 4 to 15 (Figure 1B).

A chromosomal distribution map of *StSnRK2* genes was generated. A total of eight *StSnRK2* genes were found to be unevenly distributed on 6 of 12 potato chromosomes (Figure S2). Among of them, Chr1 (*StSnRK2.3* and *StSnRK2.4*) and Chr4 (*StSnRK2.1* and *StSnRK2.5*) contain two *StSnRK2* genes each, and the rest of the chromosomes contain one *StSnRK2* gene each. Interestingly, the distribution of *StSnRK2* genes on the chromosomes is mostly concentrated at the two ends of the chromosomes, and very few *StSnRK2* genes are distributed in the middle position.

### 2.2. Phylogenetic Analysis Divides StSnRK2s into Three Subgroups

**Figure 2:**
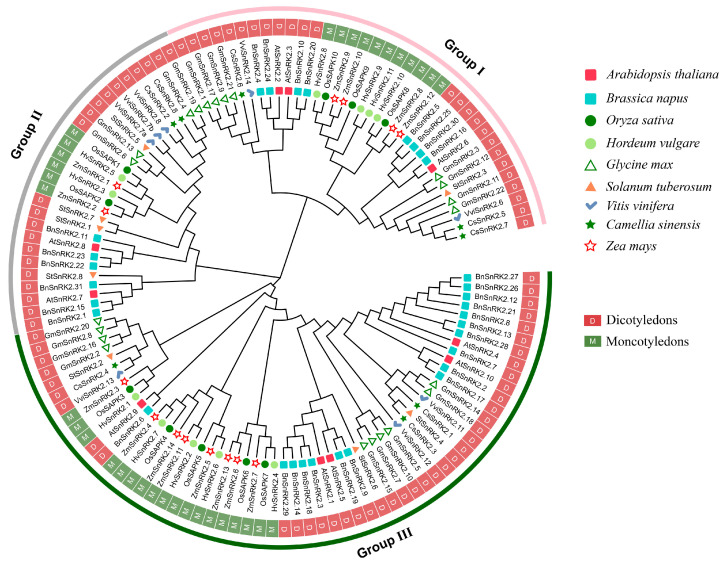
Unrooted phylogenetic tree representing the relationships among 8 StSnRK2 proteins from Solanum tuberosum and 114 SnRK2 proteins from three monocots and five dicots. The phylogenetic trees were constructed using the neighbor-joining (NJ) method in MEGA7.0, and the default parameter value was set to 1000.

In addition to StSnRK2 proteins, 114 SnRK2 protein sequences from eight species (three monocotyledons and five dicotyledons) were used to construct a phylogenetic tree, together with StSnRK2s. The tree shows that the SnRK2 proteins can be divided into three subgroups, namely group I–group III, containing 36, 27 and 59 SnRK2 proteins, respectively (Figure 2). Only 1 StSnRK2 (2.3) is classified into group I, with four StSnRK2s (2.1, 2.5, 2.7 and 2.8) classified into group II and three StSnRK2s (2.2, 2.4 and 2.6) classified into group III. Interestingly, phylogenetic tree analysis revealed that 122 SnRK2 proteins can be clearly divided into four monocotyledon and five dicotyledon subgroups. Group I and group II comprise two monocotyledon subgroups each. Group III comprises one monocotyledon subgroup.

### 2.3. Synteny Analysis of StSnRK2 Genes

**Figure 3:**
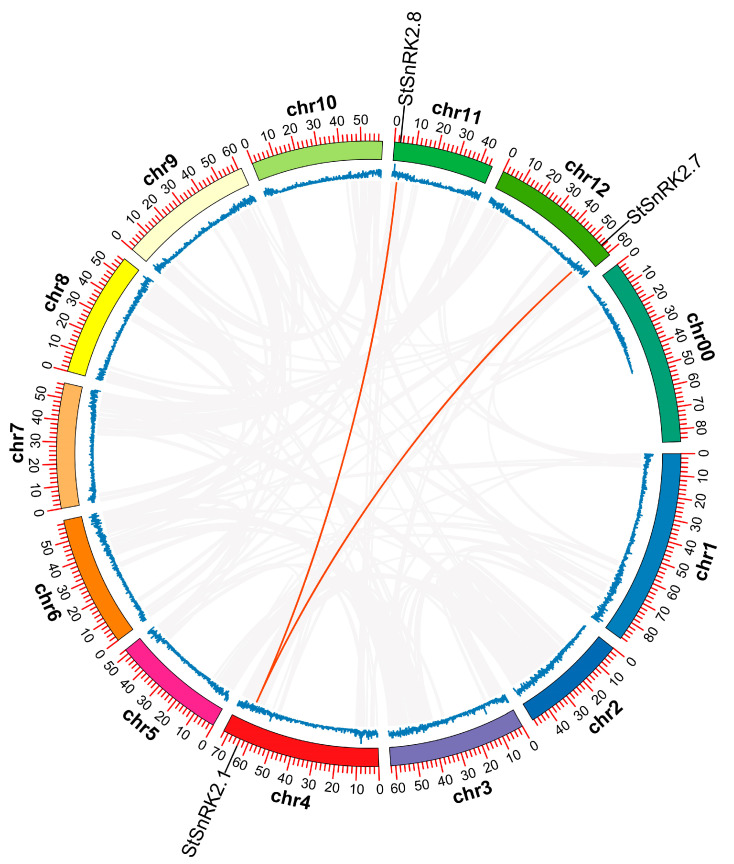
The relationship between chromosomes of *StSnRK2* from potato was determined using multiple collinear scanning toolkits (MCScanX) was visualized using TBtools v1.098669. Gray lines represent the collinear blocks within the potato genome, whereas the red lines highlight the syntenic *SnRK2* gene pairs. The height and position of the blue wave peak represent the number and distribution of genes on each chromosome, respectively.

Gene duplication plays an important role in the occurrence of new functions and the expansion of gene families. Therefore, we further analyzed the duplication events of the *StSnRK2* genes, including tandem and segmental duplication events. A chromosomal region in which two or more genes occur within 200 Kb is defined as a tandem duplication event [39]. The results show that the duplicate gene of *StSnRK2* was not detected in potato. However, two pairs of segmental duplication genes were detected between three chromosomes: Chr4 (*StSnRK2.1*)/Chr11 (*StSnRK2.8*) and Chr4 (*StSnRK2.1*)/Chr12 (*StSnRK2.7*) (Figure 3). In short, it is possible that some *StSnRK2* genes arose through segmental duplication and that these segmental duplication events were the main drivers of *StSnRK2* evolution.

**Figure 4:**
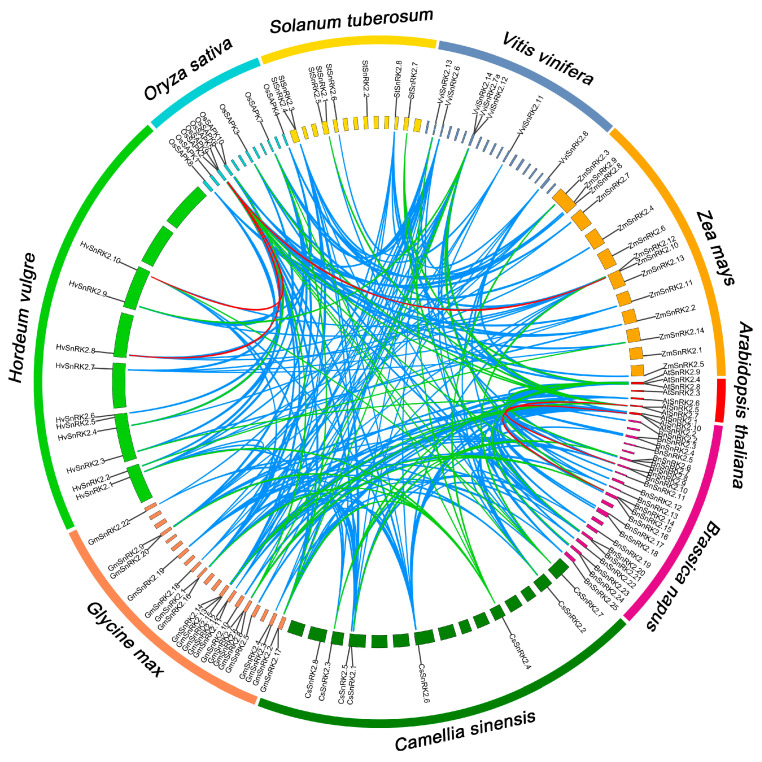
Synteny analysis among 122 *SnRK2* genes. The synteny relationship between each pair of SnRK2 genes was detected using linear regression. Lines of different colors represent the similarity of sequences: red, > 95%; 75% = blue ≤ 95%; green, < 75%.

In addition, analysis of gene duplication events showed that most *SnRK2* genes exhibited obvious duplication among nine species. A total of 122 homologous *SnRK2* genes were found in monocots and dicots. Among them, five pairs (*AtSnRK2.2*/*BnSnRK2.9*, *AtSnRK2.5*/*BnSnRK2.15*, *HvSnRK2.10*/*OsSAPK5*, *HvSnRK2.8*/*OsSAPK9* and *OsSAPK9*/*ZmSnRK2.10*) showed more than 95% similarity. An additional 37 pairs of *SnRK2* genes have a similarity of less than 75%, and 178 pairs have similarities between 75% and 95%. There were notable similarities between *StSnRK2s* and 23 *SnRK2s* that distinguished them from other species, including five in grapes, three in Arabidopsis, six in soybeans, six in camellias and four in rape (Figure 4). Interestingly, these genes that have a collinear relationship with *StSnRK2* are all from dicotyledons. These results imply that *SnRK2* genes have a wide range of duplication events in some species and that *StSnRK2s* are closer to dicotyledons during the process of evolution.

**Figure 5:**
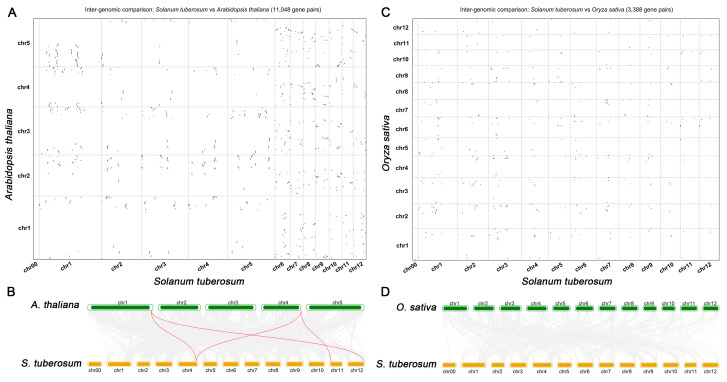
Analysis of synteny relationships. (**A**,**C**) Dot plots representations of the intergenomic comparison relationships between potato and other species. The dots represent synteny genes, and the abscissa and ordinate represent the number of chromosomes of different species. (**B**,**D**) Synteny analysis between potato and other species of the *SnRK2* gene family. Two species (Arabidopsis and rice) were compared with potato *SnRK2* genes. Gray lines represent the collinear blocks within the genomes, whereas the red lines highlight the syntenic *SnRK2* gene pairs.

To further explore the evolutionary relationships of *StSnRK2* genes, intergenomic comparison relationships maps between potato and two representative species (a dicot (Arabidopsis) and a monocot (rice)) were constructed. The results show that 11,048 homologous gene pairs were detected in potato and Arabidopsis, and the distribution shows a certain regularity. In Arabidopsis, most genes are located at the front or end of the chromosome, and few genes are located at the middle segment. In potato, homologous genes are uniformly distributed in chromosomes, except Chr00 (Figure 5A). Further analysis of the collinearity of *SnRK2* genes in potato and Arabidopsis showed that there are five collinearity gene pairs (Figure 5B). Similarly, we also analyzed the collinear relationship between potato and rice. The results show that 3388 homologous gene pairs were detected, the distribution on their chromosomes exhibits no obvious regularity (Figure 5C) and there are no collinearity gene pairs (Figure 5D). These results suggest that potato may have a distant evolutionary relationship with monocots and a close evolutionary relationship with dicots.

### 2.4. Evolutionary Selection Pressure and Codon Usage Bias Analysis 

In order to explore the evolutionary selection pressure of *StSnRK2s* between monocots and dicots, we calculated Ka, Ks and Ka/Ks among selected species on the basis of collinearity analysis. Ka and Ks are molecular evolution rates. In this study, Ka ranged from 0.05 to 0.25, and Ks ranged from 0.51 to 6.77. The Ks between *Arabidopsis thaliana* and *Brassica napus* was the smallest, and that between *Vitis vinifera* and *Zea mays* was the largest. Ka/Ks is important for assessing the selection pressure of species in evolution. The results show that all Ka/Ks values were less than 1. Therefore, the evolutionary selection pressure among the nine species exhibits a purifying selection relationship. However, the Ka/Ks among three monocots ranged from 0.08 to 0.10 (Table 1), whereas that in dicots ranged from 0.01 to 0.06 (Table 1). Moreover, the divergence time of the three monocots was similar, at 22 million years ago (Mya). However, there was a large variation among dicots, ranging from 74.70 Mya (*Camellia sinensis* and *Hordeum vulgare*) to 225.53 Mya (*Vitis vinifera* and *Zea mays*). The relative divergence time between potato and dicots is between 48.96 Mya and 81.79 Mya (Table 1). Thus, it can be concluded that the genetic relationship of the *SnRK2* gene family is relatively close among the three monocots, with more distant relationships between monocots and dicots, followed by those between dicots and dicots.

Codon usage bias analysis can reveal the genetic evolution rules between species and gene families. To understand the regulatory mechanism of transcription and translation, we analyzed related parameters such as codon adaptation index (CAI), codon bias index (CBI), frequency of optimal codons (FOP) and effective number of codons (Nc). The codon usage parameters analysis of monocots and dicots showed that the average values of synonymous codon-corresponding base frequency (C3s, G3s and GC3s), CBI and Fop were significantly higher in monocots, whereas T3s, A3s and CAI were significantly higher in dicots. In addition, the CBI of *Camellia sinensis* was considerably lower among the dicots (Table 2). Moreover, correlation analysis of codon usage parameters showed that there were certain similarities between monocots and dicots; for example, C3s and GC3s positively correlated with CAI, CBI and Fop in dicots and monocots, whereas A3s negatively correlated with CAI, CBI and Fop in dicots and monocots. Although codon preferences of monocots and dicots were obviously similar, there were also some differences. For example, G3s, C3s and GC3s negatively correlated with Nc in monocots but positively correlated with Nc in dicots, whereas T3s and Nc correlated positively in monocots but negatively in dicots (Table 3 and Table 4).

### 2.5. Prediction of Cis-Acting Elements and Binding Proteins of StSnRK2 Gene Promoters

**Figure 6:**
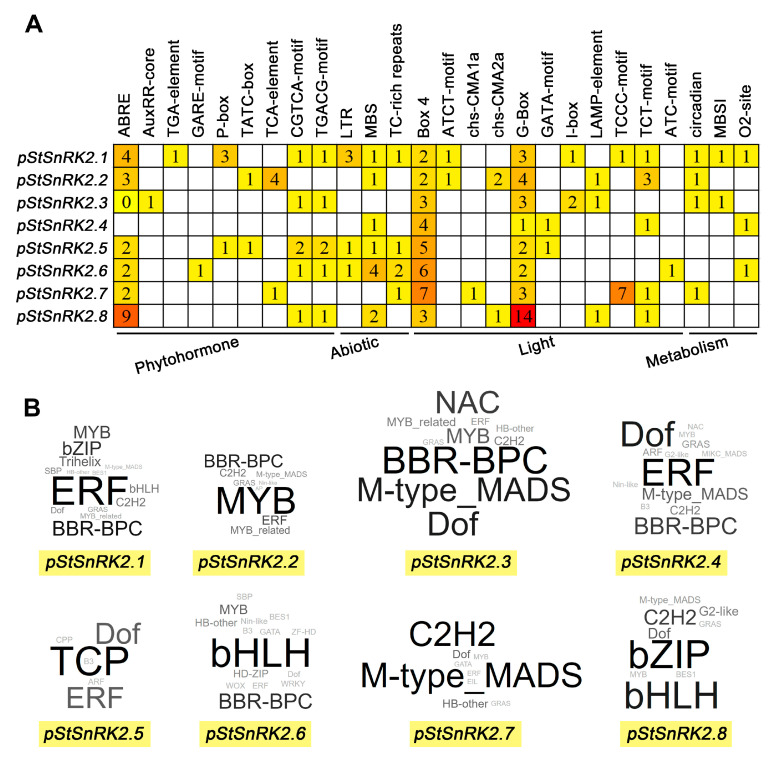
Predictive analysis of *StSnRK2* gene promoter sequences. (**A**) The types and numbers of cis elements of *StSnRK2* gene promoter sequences were analyzed by PlantCARE software. (**B**) Prediction of upstream regulatory proteins of the *StSnRK2* gene. Different words represent different protein families. The word size represents the predicted frequency of the family of proteins.

*Cis*-acting elements play a crucial role in the transcription and expression of genes and can provide a variety of functions to regulate plant growth and its adaptation to the environment [40]. To further reveal the characteristics of *StSnRK2s* and predict the possible regulatory pathways involved, the types and numbers of elements in *StSnRK2* promoter sequences were analyzed (Figure 6A). Here, we mainly focused on the environmental response elements. *StSnRK2* promoters include hormone response elements (ABRE, TGACG motif, CGTGA motif, etc.), stress response elements (MBS, LTR, TC-rich repeats, etc.) metabolism-related response elements (MBSI and O2 site) and light response elements (G-box, Box 4, TCCC motif, etc.). Among them, the number of light response elements is the largest (94), followed by ABA response elements (ABRE, 22) and drought-inducible elements (MBS, 10), with the fewest auxin response elements (AuxRR-core and TGA element, 1) and gibberellin response elements (GARE motif, 1). The hormone response elements are the most widely distributed among the promoters of *StGH3s*; for example, ABRE element exists in all *StSnRK2* gene promoters except *StSnRK2.4*. In addition, the promoter of the *StSnRK2.1* gene contains the most elements (17), whereas the promoter of the *StSnRK2.4* gene contains the fewest (6).

We also performed predictive analyses of proteins that may regulate the expression of *StSnRK2s*. The results show that 29 protein families were predicted to be involved in the regulation of *StSnRK2* gene expression (Figure 6B). *StSnRK2*.6 promoter had the largest number of predicated protein types (15), whereas *StSnRK2*.5 had the fewest (6). Among them, Dof, ERF and MYB have the highest frequency and were predicted in seven *StSnRK2* genes, followed by C2H2, GRAS and M-type_MADS in six *StSnRK2* genes. In addition, ten proteins (Trihelix, AP2, MIKC_MADS, CPP, TCP, HD-ZIP, WOX, WRKY, ZF-HD and EIL) were predicted only once, indicating that these proteins may be specific regulators of some *StSnRK2* genes. Interestingly, we found that the most frequent protein only in the prediction of *StSnRK2.1* and *StSnRK2.4* promoters was the same (ERF), whereas the most frequent proteins in the remaining *StSnRK2s* were different. These results indicate that the function and regulatory mechanism of *StSnRK2* family genes may be different in potato response to stress; therefore, further studies are needed on the functions of this family of genes in stress response.

### 2.6. Expression Patterns of StSnRK2 Genes under ABA and Drought Treatments

**Figure 7:**
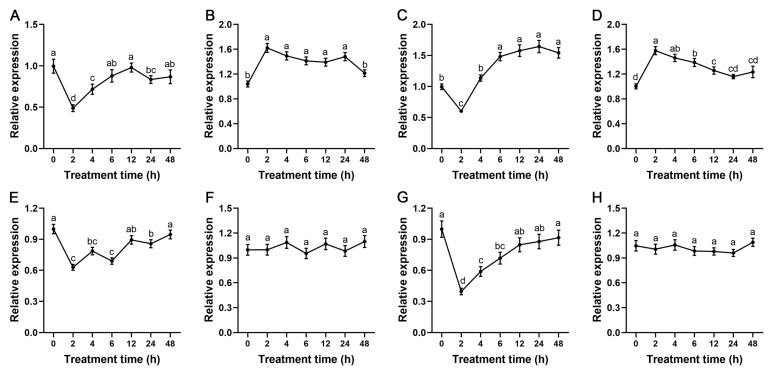
The relative expression level of *StSnRK2* genes under 50 µM ABA stress treatments. Data represent the means ± SD of three replicates, and different letters indicate significant differences at *p* < 0.05. (**A**–**H**) *StSnRK2.1*–*StSnRK2.8*, respectively.

Before studying the function of *StSnRK2* genes, we first analyzed the expression patterns of eight *StSnRK2* genes under ABA and drought stress. The qPCR results show that all the eight *StSnRK2* genes responded to stress, but the response profiles were different. Under ABA treatment (Figure 7), the expression levels of *StSnRK2.1*, *StSnRK2.3*, *StSnRK2.5* and *StSnRK2.6* genes showed a similar trend, which decreased significantly after 2 h of treatment, then rose gradually after 4 h and reached the maximum at 48 h. *StSnRK2.2* and *StSnRK2.4* reached a peak at 2 h, which was about 1.6 times that of the control, then declined gradually after 4 h and almost recovered to the level before treatment after 48 h. although there were some fluctuations in *StSnRK2.6* and *StSnRK2.8* during the whole treatment process, they did not reach significant levels, possibly indicating that they have different signaling pathways.

**Figure 8:**
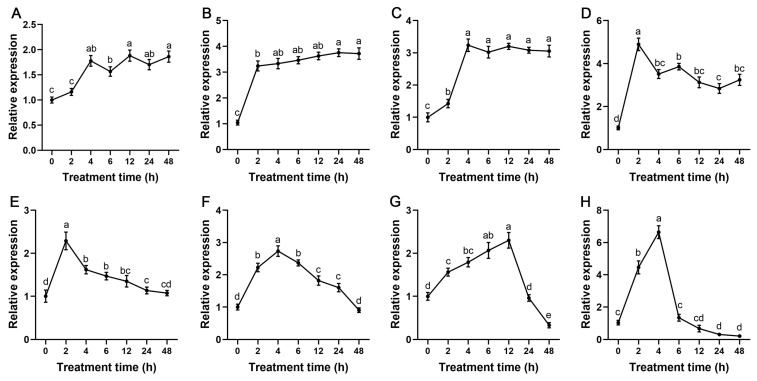
Relative expression level of *StSnRK2* genes under 5% PEG stress treatments. Data represent the means ± SD of three replicates, and different letters indicate significant differences at *p* < 0.05. (**A**–**H**) *StSnRK2.1–StSnRK2.8*, respectively.

Under drought treatment, the expression level of *StSnRK2.1*, *StSnRK2.2* and *StSnRK2.3* increased rapidly from 2 to 4 h of stress and was maintained at a relatively stable level until 48 h of treatment. The expression levels of *StSnRK2.4* and *StSnRK2.5* reached a peak at the 2 h time point, which is about 5 times as much as that of the control, then declined significantly. Expression levels of *StSnRK2.6* and *StSnRK2.7* decreased steadily with the extension of treatment time, and *StSnRK2.7* declined significantly after 24 h and 48 h compared to the control. The expression of *StSnRK2.8* was approximately seven times higher than that of the control after 4 h, then decreased gradually after 6 h and reached the minimum at 48 h (Figure 8).

### 2.7. StSnRK2 Gene Expression and Physiological Changes under Different Degrees of Drought Stress

**Figure 9:**
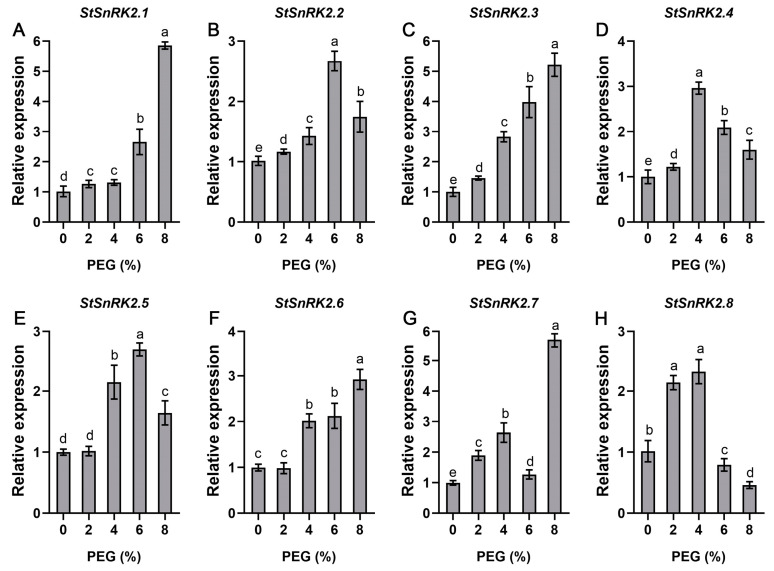
Relative expression level of *StSnRK2* genes under different concentrations of PEG stress treatments. Data represent the means ± SD of three replicates, and different letters indicate significant differences at *p* < 0.05. (**A**–**H**) *StSnRK2.1*–*StSnRK2.8*, respectively.

In order to fully understand the response of *StSnRK2* genes to drought stress, potato plantlet stems with at least two leaves were treated with different concentrations of PEG, and *StSnRK2* gene expression was detected 4 weeks later. The results show that all the genes responded to PEG, and the response levels of most genes increased significantly with increasing PEG concentrations (Figure 9). The expression of *StSnRK2.1*, *StSnRK2.3* and *StSnRK2.6* rose significantly with increased PEG concentration, and the highest expression was detected under 8% PEG stress: 5.8, 5.2 and 2.9 times higher than that of the control, respectively. The expression of *StSnRK2.2* and *StSnRK2.5* peaked at 6% concentration and decreased to a certain extent at 8%, suggesting that a high concentration of PEG may inhibit the expression of both genes. *StSnRK2.4* and *StSnRK2.8* expression increased when mild drought was applied and decreased under extreme drought conditions.

**Figure 10:**
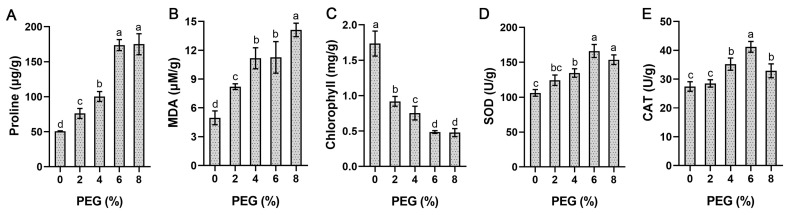
Determination of stress-related physiological indices under different concentrations of PEG stress treatments. Data represent the means ± SD of three replicates, and different letters indicate significant differences at *p* < 0.05. (**A**–**E**) Proline content, MDA content, chlorophyll content, SOD activity and CAT activity, respectively.

We also measured a number of stress-related physiological indices under the same conditions, including the content of proline, MDA and chlorophyll, as well as the activity of SOD and CAT enzymes. The results show that these physiological indices changed significantly with increased stress concentration (Figure 10). The accumulation of proline increased remarkably with increased PEG concentration, reaching a peak at 6% concentration, which was 3.4 times higher than that of the control. The change trend of MDA was similar to that of proline, but its content reached the maximum at 8% concentration. The chlorophyll content decreased drastically at 2% concentration, which was only 52% of the control, and reached a minimum value at 6% concentration. The activities of SOD and CAT first increased at and then decreased with an increase in PEG concentration and reached the maximum at 6% concentration.

Furthermore, the correlation between *StSnRK2* gene expression and changes in physiological indices was analyzed. The results show that most *StSnRK2* genes positively correlated with at least one physiological index (Table S1). In total, eight pairs of *StSnRK2* genes and physiological indices correlated significantly, of which four pairs (*StSnRK2.1* and proline, *StSnRK2.1* and MDA, *StSnRK2.1* and proline, and *StSnRK2.4* and MDA) positively correlated at the *p* < 0.01 level and four pairs (*StSnRK2.3* and SOD, *StSnRK2.4* and CAT, *StSnRK2.6* and proline, and *StSnRK2.7* and proline) positively correlated at the *p* < 0.05 level. Interestingly, we found that all *StSnRK2* genes negatively correlated with chlorophyll content, but there was no significant difference.

### 2.8. Response of StSnRK2 Genes to Drought Stress in Different Drought-Resistant Cultivars

**Figure 11:**
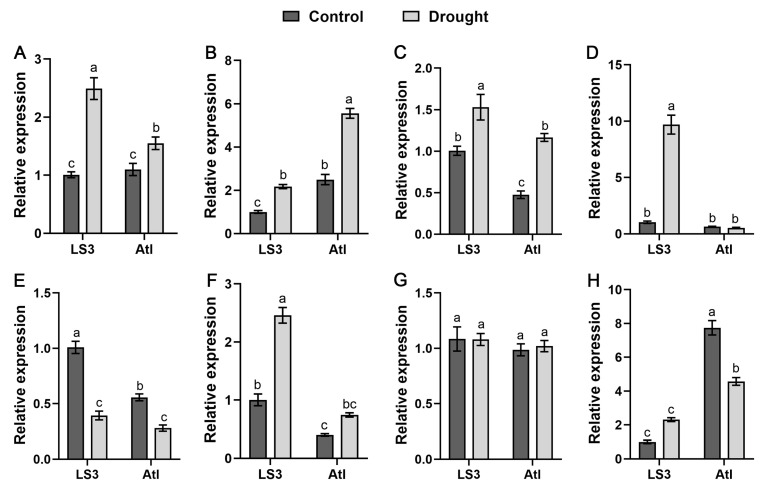
Relative expression level of *StSnRK2* genes in different drought-resistant potato cultivars (‘LS3’, drought-tolerant; ‘Atl’, drought-sensitive) under water deficit conditions. Data represent the means ± SD of three replicates, and different letters indicate significant differences at *p* < 0.05. (**A**–**H**) *StSnRK2.1–StSnRK2.8*, respectively.

In order to truly reflect the response of *StSnRK2* genes to drought stress, we further analyzed the expression of *StSnRK2* gene in two potatoes cultivars (‘LS3’, drought-tolerant; ‘Atl’, drought-sensitive) with different drought-resistance capacities under natural drought conditions. The results show that there were significant differences in the expression of eight *StSnRK2* genes between the two cultivars (Figure 11). The expression of *StSnRK2.1*, *StSnRK2.2*, *StSnRK2.3* and *StSnRK2.6* in the two cultivars after stress was significantly higher than that of the control, but there were differences in gene expression. For example, the expression of *StSnRK2.1* in ‘LS3’ was 2.5-fold higher than that of the control, whereas that in ‘Atl’ was 1.4-fold higher than that of the control. The expression of *StSnRK2.2* and *StSnRK2.6* in the two cultivars increased by about two times as much as that of the control. *StSnRK2.4* in ‘LS3’ was 9.5-fold higher than that of the control, but there was no significant change in ‘Atl’. However, *StSnRK2.5* showed a significant downward trend in both cultivars. In addition, there was no significant difference in *StSnRK2.7* expression between ‘LS3’ and ‘Atl’ before and after stress, suggesting that *StSnRK2.7* may not be directly related to drought resistance in potato.

We also measured a number of stress-related physiological indices under the same conditions (Figure S3). Proline content under normal conditions was higher in ‘LS3’ than ‘Atl’, with a similar trend observed under drought stress conditions. Proline accumulation in ‘LS3’ and ‘Atl’ was 58.5% and 46.4%, respectively, indicating that proline was more active in drought-resistant potato cultivars. Under drought stress, the antioxidant enzyme activity in both cultivars was higher compared to under unstressed treatment. The activity of SOD in ‘LS3’ and ‘Atl’ increased by 55.7% and 40.7%, respectively; CAT activity increased by 43.5% and 36.4%, respectively; and POD activity increased by 43.5% and 62.1%, respectively. Atl recorded higher MDA production under drought stress than ‘LS3’. These results suggest that SOD and CAT were more active in cultivars with strong drought resistance. 

Furthermore, the correlation between *StSnRK2* gene expression and changes in physiological indices was analyzed. The results show that most *StSnRK2* genes were markedly associated with at least one physiological index (Table S2). In total, 21 pairs of *StSnRK2* genes and physiological indices were significantly correlated, of which 18 pairs (*StSnRK2.1* and proline/MDA/SOD/POD, *StSnRK2.2* and MDA/IL/CAT, *StSnRK2.3* and proline/MDA/SOD/POD, *StSnRK2.4* and proline/SOD/POD, and *StSnRK2.6* and proline/SOD/POD) recorded positive correlations and 3 pairs (*StSnRK2.5* and MDA/IL and *StSnRK2.2* and proline) were negatively correlated. Interestingly, we found that *StSnRK2.7* has no obvious correlation with any of the analyzed physiological indices.

### 2.9. Effect of StSnRK2s on Drought Tolerance of Transgenic Tobacco

**Figure 12:**
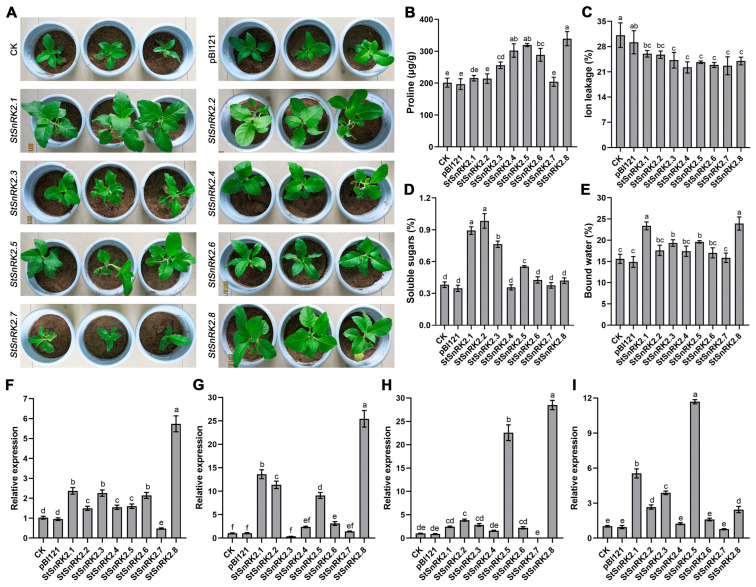
Functional identification of *StSnRK2* genes in transgenic tobacco. (**A**) Growth phenotypes of *StSnRK2* transgenic plants and control plants under drought stress. (**B**–**E**) Determination of proline content, ion leakage, soluble sugar and bound water content in transgenic plants and control plants under drought stress. (**F**–**I**) The expression levels of *NtCBL3*, *NtERD10A*, *NtERD10B* and *NtERD10C* genes in transgenic plants and wild-type plants under drought stress. Different letters indicate significant difference at *p* < 0.05.

We further overexpressed *StSnRK2* genes in tobacco to verify their biological function. First, obtained positive transgenic plants through Kana resistance screening and PCR identification (Figure S4). Then, the expression level of *StSnRK2* genes in each line was analyzed, and lines with high expression of *StSnRK2* genes were selected for subsequent experiments. Under normal conditions, there was no significant difference in growth phenotype between *StSnRK2s* transgenic plants and control plants (Figure S5). Then, 20-day-old plants were selected and exposed to drought stress. During the early stage of drought stress, the phenotypic difference among the different lines was not obvious, and the growth was basically the same. After two weeks of stress, there was a remarkably difference between the transgenic plants and the control plants. The growth of *StSnRK2.7* transgenic plants and control plants became weaker after water restriction. Although their leaf number was similar to that of other transgenic plants, the leaves became smaller and stunted in growth. However, *StSnRK2.1*, *StSnRK2.2*, *StSnRK2.5* and *StSnRK2.8* transgenic plants had a good growth phenotype under drought stress, with hypertrophic leaves and little influence of water deficit, showing strong tolerance to drought stress. *StSnRK2.4* and *StSnRK2.6* transgenic plants also showed a certain tolerance to drought, although it was significantly weaker than that of the above four genes. *StSnRK2.3* had little effect on the drought resistance of transgenic plants, and the growth phenotype of transgenic plants under drought stress was basically the same as that of the control plants. In general, overexpression of *StSnRK2* genes affected the drought resistance of transgenic tobacco to varying degrees, and the drought resistance was ranked in the order of *StSnRK2.1/2.8* > *StSnRK2.2/2.5* > *StSnRK2.4/2.6* > *StSnRK2.3* > *StSnRK2.7* (Figure 12A).

### 2.10. Physiological Characteristics of StSnRK2 Transgenic Plants under Drought Stress

To clarify the effect of *StSnRK2s* on physiological indices of transgenic plants after withholding water, we measured proline content, IL, soluble sugars and bound water. The results show that overexpression of *StSnRK2.1*, *StSnRK2.2* and *StSnRK2.7* genes had no effect on proline accumulation in transgenic plants (Figure 12B). However, the proline content of the other five transgenic plants was remarkably higher than that of the control plants, especially *StSnRK2.4*, *StSnRK2.5* and *StSnRK2.8*, which increased by 51.7%, 60.4% and 70.4%, respectively. Ion leakage is usually used to evaluate damage to plant cells under stress. The results show that the IL of eight *StSnRK2* transgenic plants under stress was significantly lower compared to than that of the control plants (Figure 12C), indicating that the *StSnRK2s* transgenic plant cells were less damaged under drought stress. In addition, we also detected soluble sugars and bound water. The overexpression of *StSnRK2.1*, *StSnRK2.2*, *StSnRK2.3* and *StSnRK2.5* significantly increased soluble sugar content in transgenic plants by 145.1%, 170.1%, 109.6% and 51.9%, respectively. However, the other four *StSnRK2* genes did not affect the accumulation of soluble sugar in transgenic plants (Figure 12D). The content of bound water in plants is another positive indicator to evaluate plant stress resistance. The accumulation of bound water in *StSnRK2.1* and *StSnRK2.8* transgenic plant leaves was significantly higher than that in control plants under stress treatment, followed *StSnRK2.3* and *StSnRK2.5* transgenic plants; *StSnRK2.2*, *StSnRK2.4* and *StSnRK2.6* were slightly higher than the control plants, but the differences were not significant (Figure 12E). 

To delve deeper into the molecular mechanism of *StSnRK2s* in drought stress, the expression levels of four genes were detected in the transgenic and control plants under drought conditions. Overexpression of *StSnRK2s* notably increased the expression level of *NtCBL3* in transgenic plants, particularly in *StSnRK2.8* overexpression lines, whereas *StSnRK2.7* lines recorded the lowest expression (Figure 12F). Overexpression of *StSnRK2.7* reduced the expression level of *NtCBL3*, which accounted for only 46.8% of the control plants. For *NtERD10A* gene, overexpression of *StSnRK2.1*, *StSnRK2.2*, *StSnRK2.5* and *StSnRK2.8* enhanced its expression by 13.1-, 10.9-, 8.7- and 24.5-fold, respectively relative to the expression in the control plants (Figure 12G). For the *NtERD10B* gene, the overexpression of *StSnRK2.5* and *StSnRK2.8* strikingly enhanced its expression level in transgenic plants by 23.8- and 30.1-fold, respectively, relative to the control plants (Figure 12H). The expression of *NtERD10C* in transgenic plants also markedly differed between transgenic plants and control plants. The expression level of *NtERD10C* in *StSnRK2.1*, *StSnRK2.2*, *StSnRK2.3* and *StSnRK2.5* transgenic plants was 5.7-, 2.7-, 3.9- and 11.9-fold of that in the control plants, respectively (Figure 12I). There was no clear difference in the expression of *NtERD10C* among other *StSnRK2* transgenic plants.

## 3. Discussion

When exposed to harmful environments, plants can adapt to stress conditions through various morphological, physiological and molecular responses [41]. Stress-induced protein kinase phosphorylation plays a very important role in the process of plant sensing and response to such stress conditions. SnRK2s, a subfamily of Ser/Thr protein kinases, plays a key role in plant responses to stresses such as drought, high salt and low temperature [42]. As an important component in PYL-PP2C-SnRK2, the core module of the ABA signal pathway, SnRK2, regulates stomatal movement through phosphorylation of downstream proteins and ultimately affects plant stress resistance [43]. In our previous study, eight *SnRK2* genes in potato were isolated and identified, and their basic gene structure and subcellular localization were analyzed [38]. However, the molecular characteristics, evolutionary relationship and biological function of *StSnRK2* genes have not been studied to date. Here, we further studied the *StSnRK2* genes according to the abovementioned aspects.

From the perspective of phylogenetic evolution, the analysis of the primary structure of protein provides important information for the evolutionary relationships among gene families. Previous studies have shown that the *SnRK2s* in most higher plants have nine exons, although some have one (*ZmSnRK2.5*) [15], two (*SbSnRK2.8*) [44], three (*OsSAPK5*) [14], five (*AtSnRK2.8*) [45] or seven (*GmSnRK2.6*) exons [16]. Generally, there are eight introns in plant *SnRK2*, indicating that these genes are highly conserved. In this study, the number of *StSnRK2* gene introns was six (*StSnRK2.6*), seven (*StSnRK2.8*), eight (*StSnRK2.1*, *2.2*, *2.5*, *2.7*) and nine (*StSnRK2.3*, *2.4*), which is not extremely conservative. This finding suggests that *SnRK2* members may experience uneven intron deletions in dicot and monocot plant lineages, leading to changes in the size and number of introns. The lengths of the second to eighth *SnRK2s* in maize and Arabidopsis were 75, 102, 54, 93, 93, 105 and 99 bp, respectively, with similar results in potato. *StSnRK2.6* has six introns because it has an extended exon. These results indicate that *SnRK2* genes evolved structural conservation among the same subgroups and diversity among different subgroups.

Gene duplication plays a key role in promoting biological evolution by creating original genetic materials that have been modified by natural selection pressure [46]. Original duplicate genes had a dual function, and the rejection of one of these two functions led to gene loss. When duplicate events occur, each gene acquires one of the functions and optimizes it separately so as to eliminate functional conflicts and ultimately ensure the stability of duplicate genes [47]. This indicates that *SnRK2* gene duplication events represent an ancient system and that they have expanded with the amplification of genome-wide replication events in plants. In this study, by analyzing the synteny and collinearity of *StSnRK2* genes, we identified two fragment replications but no tandem replication, which supports the view that a few potato genes were duplicated in the process of ancient polyploidization. Two pairs of *SnRK2* genes (*AtSnRK2.1*/*AtSnRK2.5* and *AtSnRK2.2*/*AtSnRK2.3*) in Arabidopsis were identified to have undergone segmental duplication. In rice, segmental duplication occurred in *SAPK1/SAPK2* and *SAPK4/SAPK5* [26]. Research has shown that potato, like Arabidopsis and rice, has experienced segmental duplication events [48]. The duplication of these fragments may greatly promote the expansion and evolution of *StSnRK2s*. Therefore, it is speculated that the existence of these genes in the potato genome may contribute to structural and functional innovation so that the potato can better adapt to adverse environments. New genes help existing genes adapt to new environmental conditions, and genomic tandem duplication events accelerate the evolution of new functions of replicated genes.

In order to evaluate the evolutionary relationship of StSnRK2 proteins between potato and other species, we constructed a tree based on the alignment of their full-length protein sequences. The SnRK2 family members in higher plants were previously divided into three groups [49]. However, there are very few species in which SnRK2 proteins are divided into four subgroups, such as MpSnRK2 [50] and OsSAPK2 [51]. Evolutionary studies based on the sequences of algae [52], moss, ferns and angiosperms (including Arabidopsis and rice) show that seed plants contain all groups of SnRK2; fern SnRK2 belongs to groups 4 and 3, bryophytes contain only group 3 members [53] and SnRK2 from algae is different from that in higher plants in sequence. These results indicate a possible evolutionary pattern of plant SnRK2s. Goup3 is an ancient form, group 4 appears before and after the emergence of pteridophytes, group 2 is closely related to group 4, and group 1 appears before angiosperms in its latest form. In this study, StSnRK2 proteins were divided into three subgroups, which means that potato SnRK2 may appear before fern SnRK2. Genomic comparison of SnRK2s showed potato had higher collinearity with soybean and tea than Arabidopsis and rice. Analysis of Ka/Ks between monocots and dicots is helpful to understand the evolutionary relationship between different species [54]. The Ka/Ks value has a closer evolutionary relationship among soybean, potato and rape. In addition, the occurrence probabilities of G3s, C3s and GC3s in monocotyledons are higher than those in T3s and A3s, but the results in dicotyledons are opposite.

Several studies have shown that the intensity of gene expression is usually related to differences in its promoter region. *Cis* elements in the promoter play a key role in gene response to environmental changes [55]. The analysis of promoter in this study showed that the *StSnRK2* promoters contain various types of cis elements, such as ABRE, MBS, Box4, etc. Most *StSnRK2* gene promoters contain at least such cis element, indicating that *StSnRK2s* can respond to multiple abiotic stresses. However, the gene response to stress does not always correspond to the type of response element in its promoter region. For example, ABRE is not present in the promoters of *StSnRK2.4*, although it was induced by ABA. No MBS element was found in *StSnRK2.3* and *StSnRK2.7* promoters, but these genes were induced by PEG. Therefore, the analysis of cis elements in the promoter region can provide important clues for the study of gene function, especially for gene expression patterns under different stresses. Similar results were found in maize [56], cotton [57] and wheat [58]. The prediction of upstream regulatory proteins of *StSnRK2* genes also provides a certain reference for further clarification of their regulatory mechanism. These results indicate that *StSnRK2s* participates in different stress response signal pathways and that there may be other unknown stress-related cis elements and/or unknown mechanisms involved in the regulation of *StSnRK2* genes.

Studies on the function of *SAPKs/SnRK2* have shown that this gene family play an important regulatory role in plant responses to abiotic stress. For instance, overexpression of *TaSnRK2.4* significantly increased the resistance of transgenic plants to salt, drought and freezing [59]; the overexpression of *AtSnRK2.8* significantly improved drought resistance of plants [45]; overexpression of *SAPK4* or *SAPK6* increased salt tolerance of rice [60]; and the overexpression of *ZmSnRK2.8* saved the drought-sensitive phenotype of the *ost1* mutant and significantly improved the growth and development of Arabidopsis under stress conditions [15]. *SAPK8*, *SAPK9* and *SAPK10* are homologs of *AtSnRK2.2*, *AtSnRK2.3* and *AtSnRK2.6*, respectively, belong to subgroup III of rice and are strongly activated by ABA. Overexpression of *SAPK8*, *SAPK9* or *SAPK10* in rice resulted in delayed seed germination and seedling growth [61]. In this study, the overexpression of eight *StSnRK2* genes showed significant differences in the effects on drought resistance of transgenic plants. For example, the overexpression of *StSnRK2.1* and *StSnRK2.8* significantly enhanced the tolerance of transgenic plants to drought stress; *StSnRK2.7* had little effect on the drought resistance of transgenic plants; and under *StSnRK2.3*, as the homologous gene of *AtSnRK2.2/2.3/2.6*, the change in drought resistance of transgenic plants was also weak. Moreover, determination of physiological indices showed that there were great differences in the effects of different *StSnRK2* genes on the accumulation of downstream metabolites. According to the above results, *StSnRK2* genes play an important role in plant responses to drought stress, and there may be great differences in their regulatory mechanisms. However, the present study only preliminarily verified the drought resistance of *StSnRK2* genes in model plants; whether these genes may also have a regulatory function under other stress conditions, such as high salt, low temperature or high temperature, as well as the molecular mechanism of their role, remains unclear, which will be the focus of our next study.

## 4. Materials and Methods

### 4.1. Plant Materials

Potato cultivars ‘Atlantic’ and ‘Longshu No.3’ and tobacco ‘NT12’ were obtained from the College of Agronomy of Gansu Agricultural University. Potato test-tube seedlings were grown in an artificial growth room under conditions of a 16 h light/8 h dark photoperiod and 22 ± 2 °C, with 60% relative humidity. The tobacco plants were sown in a greenhouse at 25 ± 2 °C, with a 12 h light/12 h dark photoperiod and 60% relative humidity. The plant expression vector pBI121 and agrobacterium tumefaciens EHA105 were stored in our laboratory.

### 4.2. Exogenous ABA and Drought Treatments

After 20 days of planting, potato seedlings were sprayed with alcohol solution containing 50 μM of ABA, and only alcohol was used as a control treatment. For drought treatment, 20-day-old seedlings from untreated growth medium were extirpated, placed in liquid MS medium containing 5% polyethylene glycol (PEG) and harvested at different time points (0, 2, 4, 6, 12, 24 and 48 h) for gene expression analysis. Seedlings transferred to normal MS medium were used as a control. In addition, we examined the response of potato seedlings under long-term drought stress conditions. Healthy stems were excised and inserted in a test tube containing solid MS medium supplemented with PEG6000 (w/v) at different concentrations (0, 2, 4, 6 and 8%). Four weeks later, all samples were collected for gene expression analysis and physiological determination (proline, MDA, chlorophyll, SOD and CAT activity). Seedlings obtained from ‘Atlantic’ and ‘Longshu No.3’ tubers 5 days after germination were planted in 30 cm pots filled with peat soil and monitored under natural conditions. Regular watering was carried out until plants reached a maximum height of 15 cm. Unstressed plants were kept under normal irrigation at 100% field capacity, and watering was suspended for drought-stressed plants. Fifteen days later, the samples were collected for gene expression analysis and physiological determination (proline, MDA, ion leakage, POD, SOD and CAT activity). All the stress treatment experiments were repeated three times.

### 4.3. Tobacco Transformation

The full-length cDNA sequences of *StSnRK2.1-2.8* were inserted into pBI121 driven by the CaMV 35S promoter by *Xba*I and *Sac*I restriction sites and verified by restriction endonuclease digestion and sequencing. The recombinant vector pBI121-StSnRK2s and empty vector pBI121 were transformed into wild-type *Nicotiana tabacum* (NT12) by *Agrobacterium tumefaciens* strain EHA105. The tobacco leaves infected by agrobacterium EHA105 were transferred to induction medium (MS + 1.0 g/L 6-BA) and cocultured in the dark for 3 days. Then, the infected leaves were transferred to a plant incubator (12 h light/12 h dark photoperiod, 22 ± 2 °C and 60% relative humidity) for induction and differentiation of resistant buds (MS + 1.0 g/L 6-BA + 100 mg/L Kan + 500 mg/L Cef). The resistant buds were cut and transferred to elongation medium (MS + 0.1 g/L 6-BA + 100 mg/L Kan + 500 mg/L Cef) when the buds grew to approximately 1.5 cm. Then, the resistant buds were transferred to the rooting medium (1/2 MS + 1.0 mg/L NAA + 100 mg/L Kan + 500 mg/L Cef) to induce rooting when the buds grew independently and began to take root. Finally, the positive lines were selected and verified by PCR.

### 4.4. Drought Stress of Transgenic Tobacco

*StSnRK2s* transgenic tobacco, empty vector pBI121 transgenic tobacco and wild-type tobacco were cultured in a greenhouse under the following conditions: 12 h light/12 h dark photoperiod, 22 ± 2 °C and 60% relative humidity. After 20 days of growth, plants with uniform seedling performance were selected for drought treatment. For drought-stressed plants, watering was withheld for 2 weeks, whereas control plants were irrigated optimally. The morphological characteristics of each plant were observed during the stress period. In addition, 30-day-old transgenic tobacco and wild tobacco were treated with 20% PEG for 48 h, and the expression levels of drought-stress-related genes, free water and bound water, soluble sugar, relative electrical conductivity and proline content were determined. All the stress treatment experiments were repeated three times. 

### 4.5. qRT-PCR

The expression patterns of *StSnRK2* genes under different stress conditions were analyzed by qRT-PCR. The primers of *StSnRK2s* (Table S3) were designed using Primer6. The expression level of drought-related genes in transgenic tobacco was also analyzed (Table S4). Potato *EF1α* gene was used as the internal reference. The reaction system (10 μL) comprised 5 μL PrimeSTAR^®^ Max DNA polymerase of, 1 μL of primers (10 mM) and 4 μL of ddH_2_O. The reaction conditions were as follows: 95 °C, 3 min; 95 °C, 5 s; 60 °C, 30 s; 45 cycles. The PCR results were calculated by the 2^−ΔΔCT^ method. 

### 4.6. Intro/Exon Structures, conserved Motifs/Domains and Chromosomal Distribution of Potato SnRK2 Genes

The conserved motifs and domains of SnRK2 proteins were analyzed with the MEME online program (https://meme.nbcr.net/meme/intro.html, accessed on 20 August 2020) and the NCBI Batch CD-search online website (https://www.ncbi.nlm.nih.gov/Structure/bwrpsb/bwrpsb.cgi, accessed on 25 August 2020), then visualized using TBtool. The exon/intron constituents and chromosome distribution of *StSnRK2* members were also analyzed and visualized using TBtools.

### 4.7. Phylogenetic Analysis of Potato SnRK2 Proteins

The StSnRK2 protein sequences of Arabidopsis, oilseed rape, rice, soybeans, grapes, tea tree and maize were downloaded from the NCBI or EnsemalPlants database. Then, a phylogenetic tree of potato SnRK2 proteins with other SnRK2 proteins was constructed using MEGA7 software, and 1000 bootstrap tests were carried out. Finally, the phylogenetic tree of StSnRK2 proteins was modified through the EvolView online website (https://evolgenius.info//evolview-v2/#login, accessed on 15 January 2021).

### 4.8. Intraspecific/Interspecific Collinearity Analysis of Potato SnRK2 Genes

The reference genomic sequence and GFF annotation files of potato were self-aligned in TBtools to analyze the collinearity among *StSnRK2* members. Moreover, the genomic sequence and GFF annotation files of Arabidopsis, soybean, tomato, tartary buckwheat, grape and sunflower were downloaded from the EnsemblPlants database (https://plants.ensembl.org/index.html, accessed on 17 January 2021) and aligned with the potato genome sequence by TBtools. The intraspecific and interspecific collinearity results of *StSnRK2* genes were visualized by TBtools.

### 4.9. Selection Pressur, and Codon Usage Bias Analysis of Potato SnRK2 Genes

Based on the NG method, 122 *SnRK2* genes of 9 species were employed to calculate the Ka, Ks and Ka/Ks, as well as average values of each species, to analyze the evolutional selection pressure. The divergence time was calculated as T = Ks/2r, where r was assumed to be 1.5 × 10^−8^ synonymous substitutions per site and per year for dicotyledonous plants. CodonW software was used to analyze the codon usage characteristics of the *StSnRK2* gene family in monocots and dicots. The main parameters, including A3s, G3s, C3s, T3s, CAI, CBI, FOP, Nc and GC3s, were calculated by employing all CDS sequences. The correlation relationships of codon usage parameters were analyzed in monocots and dicots.

### 4.10. Cis-Element Analysis of Potato SnRK2 Genes

The promoter sequences 2000 bp upstream of the start codon of *StSnRK2* genes were extracted by TBtools and submitted to the PlantCARE website (http://bioinformatics.psb.ugent.be/webtools/plantcare/html/, accessed on 20 March 2021) to predict the type and number of cis-acting elements in the promoter sequences. Finally, the results of cis-acting elements were visualized by TBtools. PlantRegMap online software (http://plantregmap.gao-lab.org/binding_site_prediction.php, accessed on 23 March 2021) was used to predicate the upstream regulatory proteins of *StSnRK2* genes. A word cloud was generated with online software (https://kt.fkw.com/ciyun.html?isEditor=true#/, accessed on 25 March 2021). 

### 4.11. Statistical Analysis

All experiments were performed for three independent biological repeats, and at least three technical repeats were set each time. Data were analyzed using the Student’s *t* test. A *p* value of <0.05 was considered significant.

## 5. Conclusions

In summary, the gene structure, phylogeny and evolution, chromosome location and interspecific/intraspecific collinearity of potato *SnRK2* family genes were analyzed systematically for the first time in this study. The results show that eight StSnRK2 genes were distributed on six chromosomes, coding proteins were divided into three subgroups, and StSnRK2s clustered in the same subgroup had similar conserved motifs and domains. In addition, StSnRK2 has a wide range of replication events in some species, which is closer to dicots in the process of evolution. The results of selective pressure analysis show that the average Ka/Ks value of *SnRK2s* in monocots was higher than that of dicots. Further codon preference analysis showed that *SnRK2s* prefer to use C3s, G3s and GC3s in monocots, whereas T3s and A3s are preferred in dicots. Moreover, analysis of the response to drought stress showed that some *StSnRK2* genes are significantly associated with drought stress. Finally, the functional verification results in tobacco show that the overexpression of *StSnRK2* genes improved the drought resistance of transgenic tobacco to varying degrees; the order of strength was reported as *StSnRK2.1/2.8* > *StSnRK2.2/2.5* > *StSnRK2.4/2.6* > *StSnRK2.3* > *StSnRK2.7*. This study provides useful insights into the evolution and function of *StSnRK2s* and lays a foundation for further study on the molecular mechanism of *StSnRK2s* regulating potato drought resistance.

## Figures and Tables

**Table 1:** Evolutional selection pressure between monocots and monocots, monocots and dicots and dicots and dicots.

Pattern	Species 1	Species 2	Nonsynonymous Substitution Rate (Ka)	Synonymous Substitution Rate (Ks)	Selection Strength (Ka/Ks)	Evolution Relationship	Divergence Time (Mya)
Monocots and monocots	*Oryza sativa*	*Zea mays*	0.0526	0.6623	0.0798	Purifying selection	22.0782
	*Hordeum vulgare*	*Zea mays*	0.0670	0.6602	0.1006	Purifying selection	22.0071
	*Hordeum vulgare*	*Oryza sativa*	0.0687	0.6601	0.0988	Purifying selection	22.0039
Monocots and dicots	*Camellia sinensis*	*Oryza sativa*	0.1086	2.7496	0.0396	Purifying selection	91.6526
	*Camellia sinensis*	*Hordeum vulgare*	0.1096	2.2413	0.0489	Purifying selection	74.7091
	*Camellia sinensis*	*Zea mays*	0.1468	3.1925	0.0481	Purifying selection	106.4167
	*Glycine max*	*Oryza sativa*	0.1783	3.0397	0.0588	Purifying selection	101.3221
	*Glycine max*	*Hordeum vulgare*	0.1392	2.7527	0.0505	Purifying selection	91.7571
	*Glycine max*	*Zea mays*	0.1419	2.3420	0.0607	Purifying selection	78.0659
	*Vitis vinifera*	*Hordeum vulgare*	0.1593	2.5584	0.0622	Purifying selection	85.2823
	*Vitis vinifera*	*Zea mays*	0.1292	6.7659	0.0190	Purifying selection	225.5309
Dicots and dicots	*Arabidopsis thaliana*	*Brassica napus*	0.0502	0.5086	0.0992	Purifying selection	16.9549
	*Arabidopsis thaliana*	*Glycine max*	0.1121	1.9277	0.0646	Purifying selection	64.2576
	*Arabidopsis thaliana*	*Vitis vinifera*	0.1268	1.3427	0.0944	Purifying selection	44.7592
	*Arabidopsis thaliana*	*Solanum tuberosum*	0.1268	1.7212	0.1455	Purifying selection	57.3766
	*Arabidopsis thaliana*	*Camellia sinensis*	0.1160	2.5458	0.0492	Purifying selection	84.8618
	*Brassica napus*	*Glycine max*	0.1191	1.7633	0.0682	Purifying selection	58.7779
	*Brassica napus*	*Camellia sinensis*	0.1694	1.4868	0.1192	Purifying selection	49.5612
	*Brassica napus*	*Solanum tuberosum*	0.1824	2.4539	0.0750	Purifying selection	81.7989
	*Brassica napus*	*Vitis vinifera*	0.1346	1.9996	0.0674	Purifying selection	66.6560
	*Camellia sinensis*	*Glycine max*	0.1028	1.4346	0.0762	Purifying selection	47.8206
	*Camellia sinensis*	*Solanum tuberosum*	0.0885	2.0827	0.0428	Purifying selection	69.4264
	*Camellia sinensis*	*Vitis vinifera*	0.0885	1.0203	0.0979	Purifying selection	34.0108
	*Glycine max*	*Solanum tuberosum*	0.1254	1.6182	0.0885	Purifying selection	53.9403
	*Glycine max*	*Vitis vinifera*	0.0893	1.2701	0.0722	Purifying selection	42.3375
	*Solanum tuberosum*	*Vitis vinifera*	0.1327	1.4690	0.0981	Purifying selection	48.9677

**Table 2:** Analysis of the codon usage index in nine species.

Species	T3s	C3s	A3s	G3s	GC3s	CAI	CBI	FOP	Nc
*Arabidopsis thaliana*	0.373 ± 0.096	0.245 ± 0.028	0.359 ± 0.043	0.301 ± 0.071	0.416 ± 0.068	0.177 ± 0.041	−0.069 ± 0.059	0.374 ± 0.038	52.902 ± 3.802
*Brassica napus*	0.407 ± 0.037	0.277 ± 0.028	0.317 ± 0.043	0.297 ± 0.036	0.429 ± 0.040	0.223 ± 0.021	−0.006 ± 0.050	0.418 ± 0.030	53.141 ± 2.886
*Camellia sinensis*	0.395 ± 0.032	0.256 ± 0.039	0.350 ± 0.048	0.288 ± 0.033	0.409 ± 0.050	0.196 ± 0.016	−0.070 ± 0.069	0.377 ± 0.039	55.359 ± 2.575
*Glycine max*	0.434 ± 0.025	0.220 ± 0.023	0.341 ± 0.039	0.304 ± 0.036	0.386 ± 0.032	0.187 ± 0.016	−0.116 ± 0.040	0.351 ± 0.025	52.645 ± 2.653
*Hordeum vulgare*	0.229 ± 0.076	0.292 ± 0.069	0.284 ± 0.054	0.367 ± 0.067	0.556 ± 0.081	0.142 ± 0.042	−0.063 ± 0.051	0.384 ± 0.029	54.339 ± 4.296
*Oryza sativa*	0.276 ± 0.066	0.303 ± 0.092	0.317 ± 0.100	0.316 ± 0.097	0.502 ± 0.121	0.168 ± 0.056	−0.011 ± 0.086	0.406 ± 0.051	53.879 ± 2.220
*Solanum tuberosum*	0.382 ± 0.036	0.247 ± 0.019	0.415 ± 0.040	0.241 ± 0.077	0.372 ± 0.045	0.172 ± 0.020	−0.106 ± 0.031	0.362 ± 0.018	51.548 ± 1.442
*Vitis vinifera*	0.398 ± 0.028	0.231 ± 0.026	0.348 ± 0.031	0.320 ± 0.040	0.408 ± 0.043	0.176 ± 0.018	−0.137 ± 0.063	0.338 ± 0.038	55.103 ± 3.300
*Zea mays*	0.318 ± 0.085	0.297 ± 0.108	0.354 ± 0.115	0.277 ± 0.128	0.452 ± 0.162	0.178 ± 0.043	−0.042 ± 0.082	0.393 ± 0.046	52.848 ± 5.337

**Table 3:** Correlation analysis of codon usage indices in monocots.

	T3s	C3s	A3s	G3s	GC3s
CAI	0.2666 *	0.4727 *	−0.5666 *	0.2097	0.3364 *
CBI	−0.1442	0.6568 *	−0.4286 *	0.2097	0.5018 *
FOP	−0.0598	0.6568 *	−0.3592 *	0.0543	0.4095 *
Nc	−0.4185 *	0.4242 *	−0.3592 *	0.0543 *	0.4840 *

* indicate significant difference at *p* < 0.05.

**Table 4:** Correlation analysis of codon usage indices in dicots.

	T3s	C3s	A3s	G3s	GC3s
CAI	−0.0119	0.8613 *	−0.5995 *	0.4265 *	0.4933 *
CBI	−0.4263 *	0.8336 *	−0.7127 *	0.5654 *	0.6814 *
FOP	−0.4431 *	0.8667 *	−0.7600 *	0.6128 *	0.6814 *
Nc	0.5374 *	−0.3702 *	0.3275	0.6128 *	0.6814 *

* indicate significant difference at *p* < 0.05.

## Data Availability

All data are available within the manuscript.

## Supplementary Materials

The supporting information can be downloaded at: https://www.mdpi.com/article/10.3390/ijms24021000/s1.
