# Supplementary material for: Evolutionary Analysis of StSnRK2 Family Genes and Their Overexpression in Transgenic Tobacco Improve Drought Tolerance
Source: Int J Mol Sci. 2023 Jan 5;24(2):1000. doi: 10.3390/ijms24021000 (PMC9861535; doi:10.3390/ijms24021000)
Supplement: Supplementary file 1 [file ijms-24-01000-s001.zip › Revised supplementary material/Suplementary figures.pdf]

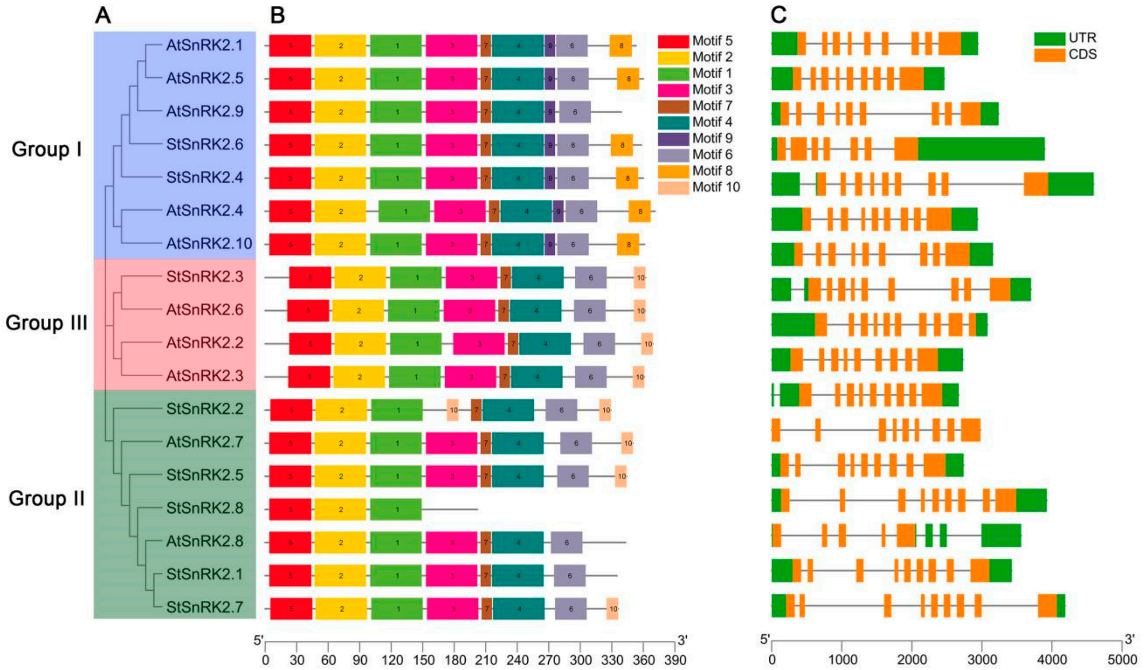

Figure S1. Phylogenetic relationships, conserved motifs and gene structure of the *StSnRK2* and *AtSnRK2* genes. (**A**) The phylogenetic tree was constructed based on the full-length sequences of SnRK2 proteins. (**B**) The motif compositions of StSnRK2 and AtSnRK2 proteins. The motifs, numbered 1-10, are displayed in different colored boxes. The protein length can be estimated using the scale at the bottom. (**C**) Exon-intron structures of *StSnRK2* and *AtSnRK2* genes. Green boxes indicate untranslated 5′- and 3′-regions; orange boxes indicate exons; and black lines indicate introns.

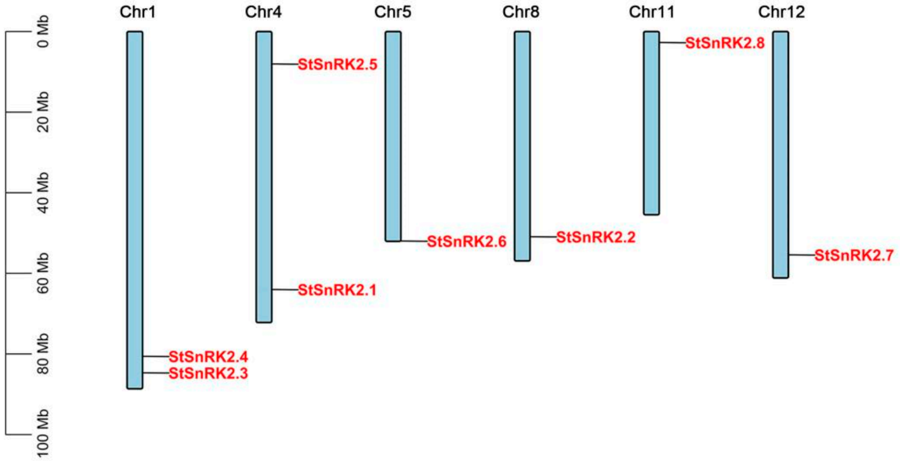

Figure S2. Schematic representations of the chromosomal distribution of the *StSnRK2* genes. The chromosome number is indicated to the left of each chromosome.

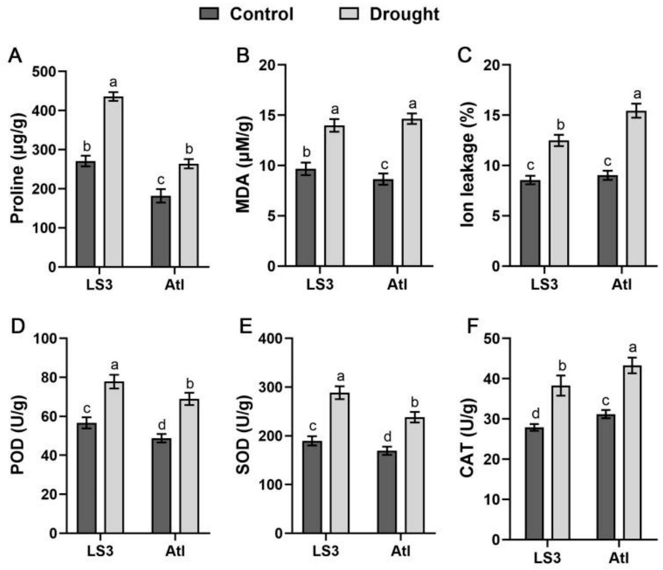

Figrue S3. Determination of stress related physiological indexes in different drought-resistant potato varieties ('LS3', drought tolerance; 'Atl', drought sensitive) under water deficit condition. Data represent the means ± SD of three replicates and different letters indicate significant difference at p<0.05. A-H represents proline content, MDA content, ion leakage, POD activity, SOD activity, CAT activity, respectively.

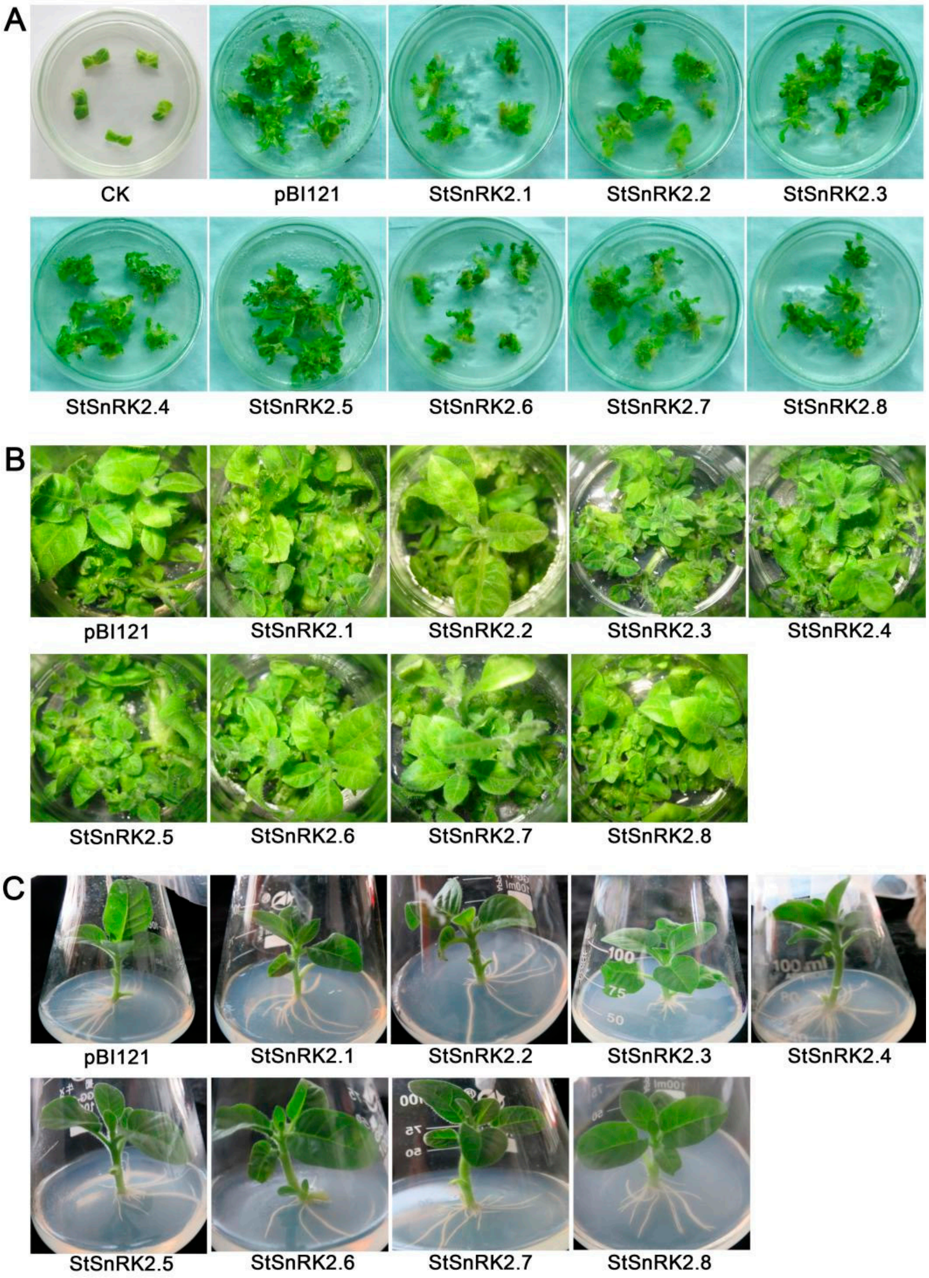

Figure S4 The screening process of *StSnRK2* transgenic tobacco. (**A**) Growth phenotype of StSnRK2 transgenic callus on Kana screening medium. (**B**) Seedlings after differentiation of SnRK2 transgenic callus. (**C**) Rooting of seedlings after differentiation of SnRK2 transgenic callus.

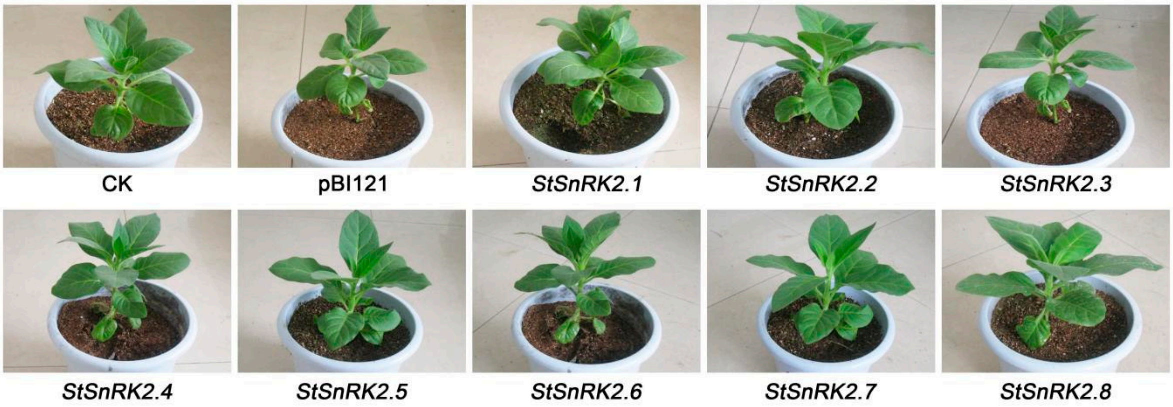

Figure S5 The growth phenotype of *StSnRK2* transgenic plants and control plants under normal conditions.
