# Supplementary material for: Evolutionary Analysis of StSnRK2 Family Genes and Their Overexpression in Transgenic Tobacco Improve Drought Tolerance
Source: Int J Mol Sci. 2023 Jan 5;24(2):1000. doi: 10.3390/ijms24021000 (PMC9861535; doi:10.3390/ijms24021000)
Supplement: Supplementary file 1 [file ijms-24-01000-s001.zip › Revised supplementary material/Suplementary tables.pdf]

Table S1. Correlation analysis of *StSnRK2* genes expression and physical properties following PEG treatment

Correlation coefficient	*StSnRK2.1*	*StSnRK2.2*	*StSnRK2.3*	*StSnRK2.4*	*StSnRK2.5*	*StSnRK2.6*	*StSnRK2.7*	*StSnRK2.8*
Chlorophyll	-0.495	-0.488	-0.418	-0.507	-0.206	-0.264	-0.447	-0.100
Proline	0.650**	0.351	0.720**	0.495	0.423	0.641*	0.609*	-0.017
MDA	0.661**	0.188	0.405	0.686**	-0.047	0.268	0.462	-0.101
CAT	0.231	0.419	0.497	0.615*	0.412	0.396	0.354	0.252
SOD	0.433	0.507	0.514*	0.494	0.395	0.370	0.443	0.188

Table S2. Correlation analysis of *StSnRK2* gene expression and physical properties following PEG treatment

Correlation coefficient	*StSnRK2.1*	*StSnRK2.2*	*StSnRK2.3*	*StSnRK2.4*	*StSnRK2.5*	*StSnRK2.6*	*StSnRK2.7*	*StSnRK2.8*
Proline	0.888**	-0.143	0.898**	0.911**	-0.249	0.958**	0.239	-0.619*
MDA	0.732**	0.623*	0.811**	0.476	-0.697*	0.534	0.071	-0.266
IL	0.552	0.855**	0.571	0.198	-0.812*	0.222	-0.021	0.007
SOD	0.941**	0.252	0.913**	0.814**	-0.571	0.840**	0.139	-0.410
POD	0.887**	0.312	0.914**	0.726**	-0.562	0.776**	0.121	0.113
CAT	0.599*	0.832**	0.55	0.270	-0.876	0.263	-0.067	0.113

Table S3 The sequences of primers were used for *StSnRK2s* in qRT-PCR

Gene	Sequences
*StSnRK*2.1	F: 5'- CAATGTGCCGATGTAAATAACCC-3'
	R: 5'- CCCATAAGAATGTGCTCCAACC-3'
*StSnRK*2.2	F: 5'-TACCTGGAGTAGTGTCA-3'
	R: 5'-AAGTCATTGCTGCTGTC-3′
*StSnRK*2.3	F: 5'-ACCAGTTTGAGGAGCCA-3'
	R: 5'-ATATCGAGGTCAGGATC-3'
*StSnRK*2.4	F: 5'-TTCCCGTTCAGTATCAG-3'
	R: 5'-TTCTTCATCCTCCTCCT-3'
*StSnRK*2.5	F: 5'- ATAGTGAAGAAAAGCCATTACAAAG-3'
	R: 5'- TCTCATCATCTAAATCAGCATCAGT-3'
*StSnRK*2.6	F: 5'-GACAATCCAACATTCTCCCTTCA-3'
	R: 5'- TGATGGAGGTGGTGGATTTCTT-3'
*StSnRK*2.7	F: 5'- GGAAGAAGGACGGAATGAGTG-3'
	R: 5'- ATGCCTTGAGATGAACTTGCT-3'
*StSnRK*2.8	F: 5'-GGCCCAAAGGTTGTGAT-3'
	R: 5'-AAATTCCCCACTTGTCT-3'

ef1a	F: 5'-CAAGGATGACCCAGCCAAG-3'
	R: 5'-TTCCTTACCTGAACGCCTGT-3'

Table S4. The sequences of primers were used for qRT-PCR in transgenic tobacco.

Gene	Sequences
NtCBL3	F: 5'- GTGGTTGCTACCCTTG-3'
	R: 5'- GCTCCATTCCTCCTTAT-3'
NtERD10A	F: 5'-GGACAAGATTAAGGAGAA-3'
	R: 5'-TGGTGGCCAGGAAG-3′
NtERD10B	F: 5'-ATGCTCCACCGTTCTG-3'
	R: 5'-TGATGATGCTCCCCTT-3'
NtERD10C	F: 5'-AGAAATACGAGGAAACAG-3'
	R: 5'-TCTTGTCCAAAAATCCC-3'
